# Teriparatide use during an economic crisis: baseline data from the Greek cohort of the Extended Forsteo Observational Study (ExFOS)

**DOI:** 10.1186/s12891-015-0600-8

**Published:** 2015-06-05

**Authors:** Kyriakos Aloumanis, George Kapetanos, Nikolaos Bartzis, Vangelis Drossinos, for the Hellenic ExFOS study group

**Affiliations:** Department of Medical Research, Pharmaserve Lilly SACI, Arkadias 1 and Megaloupoleos str, 14564 Kifissia, Athens, Greece; 3rd Orthopedics University Clinic, Papageorgiou General Hospital, Thessaloniki, Greece

**Keywords:** Osteoporosis, Teriparatide, Greece, Fracture, Back pain, Quality of life

## Abstract

**Background:**

The Extended Forsteo Observational Study (ExFOS) is a multinational, non-interventional, prospective, observational study that aims to provide real-life data on patients with osteoporosis treated with teriparatide for up to 24 months. It includes the new indications of osteoporosis in men and glucocorticoid-induced osteoporosis (GIOP). We describe the Greek subpopulation enrolled in this study and compare it with a similar cohort from the previous European Forsteo Observational Study (EFOS).

**Methods:**

Baseline data were collected from the Greek cohort of ExFOS. Data included demographic characteristics, medical and osteoporosis history, disease status, prior use of medications, back pain and quality of life.

**Results:**

Baseline data for 439 patients, enrolled at 31 sites, indicated the majority of patients were females (92.3 %), elderly [mean (standard deviation; SD) age 70.1 (9.8) years] and slightly overweight [mean (SD) body mass index 26.7 (4.3) kg/m^2^], with very low bone mineral density (mean T-score <−3 in lumbar spine or total hip) and at least one previous fracture (55.1 % of patients). Of the 439 patients, 19.8 % were osteoporosis treatment naïve, 88.4 % had experienced back pain during the previous 12 months, 68.1 % had experienced back pain at least fairly often during the previous month and 50.9 % reported moderate to severe limitation of activities due to back pain, with a mean (SD) of 4.2 (7.7) days spent in bed because of back pain during the previous month. Most baseline characteristics were numerically similar between the female ExFOS and EFOS cohorts; however, the rate of enrolment was faster in ExFOS (by approximately 45 %) and a history of fracture was recorded in 53.8 % of female patients in ExFOS versus 74.5 % in EFOS.

**Conclusions:**

Greek patients prescribed teriparatide in ExFOS had severe osteoporosis with a high risk of fractures and back pain. Female patients shared similarities with EFOS counterparts, reflecting a constant prescribing profile for use of teriparatide, although a noticeable difference in fracture history between the two study cohorts may indicate a change towards prescribing in less severely affected patients. The economic crisis in Greece did not appear to affect patient enrolment. Data are interpreted in the context of an observational setting.

## Background

Teriparatide is a well-established anabolic treatment for osteoporosis [1, 2]. Numerous studies have provided evidence of its efficacy in terms of bone density improvement and reductions in the risk of fracture [3–10]. Among these, the European Forsteo Observational Study (EFOS) has recorded the benefits of teriparatide use in real life, in reducing the risk of fracture, alleviating back pain and improving quality of life, when used for up to 18 consecutive months by postmenopausal women with osteoporosis [4, 5, 11]. Greece was one of the eight European countries that participated in this study, enrolling 302 (18.4 %) of the 1645 post-menopausal women with severe osteoporosis and considerable health-related problems [12, 13]. Teriparatide treatment was associated with significant improvements in Greek patients’ quality of life and back pain, together with reduced fracture rates and a high rate of treatment compliance [11].

Randomised controlled trials are the gold standard for treatment evaluation, yet they often under-represent the patient population in real life. Observational studies can provide valuable information that complements results obtained from randomised controlled trials and extends knowledge, identifying clinically important differences between therapeutic options [14]. In this context, results from EFOS have made an important contribution to the accumulating knowledge concerning teriparatide. Since EFOS, teriparatide has been licensed for the treatment of osteoporosis in men at increased risk of fracture, for the treatment of glucocorticoid-induced osteoporosis (GIOP) in men and women at increased risk of fractures, and the potential duration of teriparatide therapy has been extended to up to 24 months in the European Union [15].

The economic crisis in Greece has had a deteriorating impact on health care provision in the country [16, 17]. While there are data to support the cost-effectiveness of teriparatide [18], health-economic studies have not been conducted in Greece. Patients are eligible for treatment with teriparatide only after case review by special health committees and once all criteria for reimbursement have been fulfilled. In this economic arena, at least 5 years after EFOS, Greece participated in the Extended Forsteo Observational Study (ExFOS) [19]. This is a multinational, non-interventional, prospective, observational study to evaluate fracture outcomes, back pain, compliance and health-related quality of life in patients with osteoporosis treated with teriparatide, incorporating the drug’s additional indications and extended treatment duration, as described earlier. Analysis of the background characteristics of the Greek cohort from ExFOS, and comparison of the findings with those of the Greek cohort from EFOS, could give an important and intriguing insight into changes in the prescribing habits of Greek health care providers as a result of changes in the Social Security System criteria for reimbursement on prescription of teriparatide occurring amidst an economic crisis. As the Greek EFOS patients were enrolled between July 2004 and September 2005, and the Greek ExFOS patients were enrolled between February 2011 and May 2012, we additionally sought to answer the following questions: a) What may have changed during the intervening period of 5 to 6 years of teriparatide use in a national population with relative homogeneity? b) How have health economics affected prescribing of teriparatide in a country with severe financial problems? c) How have the new indications for teriparatide affected treatment choices for health care practitioners? This report describes the baseline profile of Greek patients treated with teriparatide in ExFOS, provides details by gender and juxtaposes the characteristics of female Greek patients enrolled in ExFOS with those enrolled in EFOS.

## Methods

The study was conducted in compliance with the Declaration of Helsinki and local approval was provided by the Hellenic National Organization for Medicines (NOM) [reference number: NIS-22/01-03/10, 31 January 2011]. All patients provided written informed consent before entering the study. Full details of the design and data acquisition in ExFOS have been published elsewhere [19]. In summary, baseline assessments included recording patient demographic characteristics, lifestyle and risk factors for osteoporosis and falls, prior and current osteoporosis therapies, and any accompanying diseases. In addition, the start date and reimbursement details for teriparatide treatment were noted and information on bone mineral density (BMD) was collected together with the history, number and location of any osteoporotic fractures before the start of treatment. Back pain, self-assessed by patients, was recorded. Use of analgesia for back pain in the past month and the severity of pain during the last month were also recorded. Health-related quality of life was assessed using the EuroQol-5 Dimension (EQ-5D) instrument and the related visual analogue scale (EQ-VAS).

Data management and analyses were conducted according to a pre-specified (common for all participating countries) statistical analytical plan that provided descriptive summary statistics only [mean and standard deviation (SD) for continuous data; number and percentages for categorical data]. These were used to describe the total study population of 1607 patients with osteoporosis at baseline [19].

Regarding bone mineral density, given the fact that there was no specific request per protocol, other than the investigators’ clinical practice, an analysis of obtained measurements per measurement site has been facilitated. T-scores were reported in standard deviation units relative to the reference mean BMD of a healthy young adult. When patients had several measurements at one location, the last nonmissing value before baseline was used; if several measurements at the same location and date were available, then the lowest nonmissing value was used. Since in Greece only one location measurement is reimbursed, it is probable that results of different locations may not apply to same patients (i.e. lumbar spine measurements may have been recorded from different patients than femoral neck measurements). For this reason BMD is described by the measurement result and the number (N) of measurements captured.

An additional post-hoc descriptive statistical analysis was performed using the Greek cohort to evaluate results by gender, taking into account the small size of the male subpopulation in ExFOS, and to descriptively compare results from the postmenopausal female patients in ExFOS with those from the 302 postmenopausal women in the EFOS Greek cohort included in the analyses of Karras et al. [12].

## Results

### Patients

A total of 31 investigative sites in Greece enrolled 439 patients with osteoporosis (92.3 % female) between 16 February 2011 and 24 May 2012, an enrolment period of 15 months. The mean enrolment rate was 0.95 patients/site/month (calculated by dividing the total patient number enrolled by the total number of investigational sites and the total enrolment period). Baseline characteristics of these patients are shown in Table 1, together with a gender sub-analysis. For comparison, baseline characteristics of the Greek cohort from EFOS are also included [12].

**Table 1:** Descriptive baseline characteristics of Greek patients enrolled in ExFOS and EFOS

*Greek population*	ExFOS total population	ExFOS males	ExFOS females	*EFOS* [12]
Enrolment period (dates of first and last patient enrolment)	16 Feb 2011 – 24 May 2012	//	//	*05 Jul 2004 – 30 Sep 2005*
Enrolment rate (patients/site/month)	0.95	//	//	*0.65*
Number of patients (% women)	439 (92.3)	34	405	*302 (100)*
Mean (SD) age (years)	70.1 (9.8)	71.5 (10.3)	70.0 (9.8)	*70.0 (8.5)*
Mean (SD) BMI (kg/m^2^)	26.7 (4.3)	26.4 (4.6)	26.7 (4.3)	*26.3 (4.0)*
***Menstrual history***				
Mean (SD) time since menopause (years)	-	-	22.1 (10.3)	*24.4 (9.0)*
Mean (SD) fertile period (years)	-	-	34.6 (5.6)	*32.4 (5.9)*
Early menopause before age 40 years (% patients)	-	-	5.5	*9.3*
***Fracture risk factors (% patients)***				
Current smokers	12.1	33.3	10.4	*12.6*
No exercise	49.2	61.8	48.1	*71.5*
Hip fracture in biological mother	18.1	14.8	18.4	*16.6*
One or more falls in the preceding year	37.1	50.0	36.0	*40.7*
Assist standing using arms	41.3	58.8	39.8	*92.1*
***Bone health***				
Mean (SD) DXA BMD spine	−3.4 (0.7)	−3.3 (1.3)	−3.4 (0.7)	−*3.4 (0.7)*
Mean (SD) DXA BMD total hip (higher N included)	−3.2 (0.5)	−3.4 (0.6)	−3.2 (0.5)	−*2.8 (1.1)*
Mean (SD) DXA BMD femoral neck (higher N included)	−2.8 (1.2	−3.0 (0.4)	−2.8 (1.2)	−*3.1 (0.8)*
Fracture history (% patients)	55.1	70.6	53.8	*74.5* ^a^
Mean (SD) fracture number (for patients with at least one fracture)	1.7 (1.1)	2.0 (1.9)	1.7 (1.0)	*1.8 (1.4)*
Patients with vertebral fractures (% patients)	38.7	50	37.8	*59.6* ^b^
Patients with vertebral fractures (% of patients with fractures)	70.2	70.8	70.2	*85.9*
Patients with hip fractures (% of patients with fractures)	8.3	20.8	6.9	*2.7*
***Prior treatments (% patients)***				
Prior anti-osteoporotic treatments	80.2	55.9	82.2	*83.1*
Calcitonin	23.2^c^	5.9	24.7	*64.6*
Bisphosphonates	59.0^c^	32.4	61.2	*36.1*
Prior glucocorticoid use	8.9^c^	17.6	8.1	*14.6*
***Back pain and quality of life variables***				
Back pain in the previous year (% patients)	88.4	76.5	89.4	*97.4*
Moderate to severe pain in the month before enrolment (% patients)	80.9	88.0	80.4	*85.3*
Mean (SD) EQ-5D VAS (cm)	57.0 (21.7)	56.9 (20.7)	57.0 (21.8)	*54.2 (24.9)*

^a^In EFOS, 15.9 % of patients did not have a fracture history and data were missing for 9.6 % of patients

In ExFOS, 44.9 % of patients did not have a fracture history

^b^ *Data on file*

^c^Percentage of all patients enrolled

BMD, bone mineral density BMI, body mass index; DXA, dual-energy X-ray absorptiometry; EFOS, European Forsteo Observational Study; ExFOS, Extended Forsteo Observational Study; SD, standard deviation, VAS, visual analogue scale

Greek patients enrolled in ExFOS were generally elderly [mean (SD) age 70.1 (9.8) years; 36.4 % aged 75 years or older] and slightly overweight with a mean (SD) body mass index of 26.7 (4.3) kg/m^2^. The majority of patients (92.3 %) were women, who had been post-menopausal for a mean (SD) of 22.1 (10.3) years and had experienced a mean (SD) of 34.6 (5.6) fertile years; 5.5 % reported early menopause (Table 1). Of the 405 women, 92.1 % had given birth to least one child and 30.6 % were multiparous with three or more children. Although 73.8 % of patients reported no concurrent disease, existing predisposing risk factors for fracture were fairly common: 18.1 % reported a history of maternal hip fracture, 37.1 % had experienced a fall in the last year and 37.9 % had sight problems. In addition, 49.2 % of all patients took no exercise, 12.1 % were current smokers (33.3 % of men, 10.4 % of women) and 74.8 % had never smoked. Muscle weakness, recorded as the need to use arms when standing up from a chair, was reported in 41.3 % of patients; however, only 1.6 % of patients had been immobilised for > 12 months. In addition, 69.5 % of all patients used at least one medication associated with a risk of osteoporosis or falls; specifically, current glucocorticoid use was reported by 8.9 % of patients, more commonly in men (17.6 %) than women (8.1 %). Other drugs in this category (medication related to osteoporosis and falls) included antihypertensives (47.6 %), thyroid hormones (21.2 %), antidepressants (12.3 %), antiarrythmics (9.1 %) anticoagulants or heparin (7.1 %), benzodiazepines (5.7 %), antidiabetic drugs (4.8 %) and anticonvulsants (2.1 %).

### Bone mineral density and fracture history

**Fig. 1:**
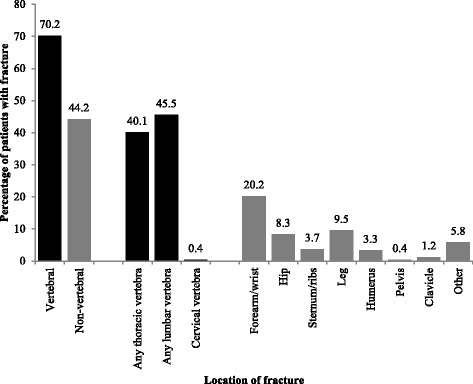
Location of fractures for Greek patients with a history of osteoporosis-related fractures (N = 242)

Table 2 depicts the distribution of BMD measurements per location. Recent (within 6 months prior to study entry) mean (SD, N) BMD T-scores were −3.4 (0.7, 280) for the lumbar spine and −3.2 (0.5, 82) to −3 (0.5, 37) for the total hip and −2.8 (1.2, 45) to −3.0 (0.5, 20) for femoral neck (left and right leg respectively). At least one osteoporotic fracture had already been diagnosed in 55.1 % of patients at baseline (70.2 % of these patients had vertebral fractures and 44.2 % had non-vertebral fractures), and 21.6 % of patients had two or more previous osteoporotic fractures. The mean (SD) number of previous fractures for patients with at least one fracture was 1.7 (1.1) [vertebral fractures 1.6 (1.0), non-vertebral fractures 1.3 (0.7)]. The most recent fracture had occurred a mean (SD) of 2.6 (5.1) years before entry into the study. Importantly, a fifth of male respondents (20.8 %) suffered a hip fracture, roughly triple the proportion of female participants (6.9 %). Figure 1 shows the location of fractures for patients with a history of osteoporosis-related fractures.

**Table 2:** Bone mineral density per measurement location, reported as percentage of measurements per location (Number of measurements)

Measured site (percentage per location and number of patients, p (N)	BMD ≤ −3.5	−3.5 < BMD ≤ −2.5	−2.5 < BMD ≤ −1	BMD > −1	Total
Lumbar spine	37.9 % (106)	55.4 % (155)	6.4 % (18)	0.4 % (1)	100 % (279)
Total Hip (left)	23.2 % (19)	70.7 % (58)	6.1 % (5)	0 % (0)	100 % (82)
Total Hip (right)	13.5 % (5)	73.0 % (27)	13.5 % (5)	0 % (0)	100 % (37)
Femoral Neck (left)	17.8 % (8)	64.4 % (29)	13.3 % (6)	4.4 % (2)	100 % (45)
Femoral Neck (right)	15.0 % (3)	80.0 % (16)	5.0 % (1)	0 % (0)	100 % (20)

### Treatment for osteoporosis

Overall, 19.8 % of Greek patients enrolled in ExFOS were osteoporosis treatment naïve at baseline; more women than men had received prior osteoporosis medication (82.2 % of women versus 55.9 % of men). Of the 352 patients treated with osteoporosis medication before enrolment, 73.6 % had used bisphosphonates and 29.0 % calcitonin, while only 10.5 % of patients had used strontium and 6.3 % raloxifene. Prior calcium and vitamin D supplementation was used by 91.5 % and 90.6 % of patients receiving osteoporosis treatment, respectively.

### Back pain

The majority of patients (88.4 %) had experienced back pain during the previous 12 months. Of the 376 patients who had back pain in the previous month, 23.7 % had experienced back pain fairly often and 44.4 % had experienced back pain every day or almost every day, the pain being moderate or severe in 80.9 % of patients. Moderate or severe limitation of activities in the past month due to back pain was reported by 50.9 % of participants, with a mean (SD) of 4.2 (7.7) days spent in bed because of back pain. Use of an analgesic medication for back pain in the month before baseline was reported by 75.7 % of the cohort. The most frequently used analgesics were paracetamol (86.8 % of those taking analgesic medication) and non-steroidal anti-inflammatory drugs/aspirin (45.7 %), with these two types of analgesic being used daily by 50.6 % and 55.0 % of patients reporting their use, respectively. The baseline mean (SD) back pain VAS score was 52.4 (29.5).

### Quality of life

**Fig. 2:**
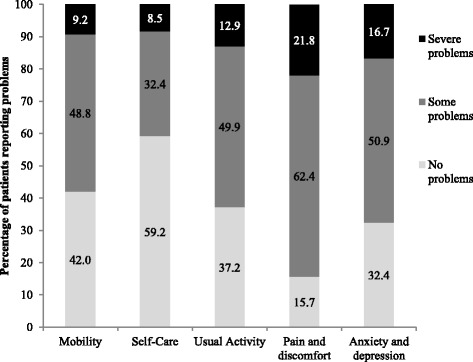
Patient quality of life at baseline according to the five EQ-5D domains. N = 426 for each domain except Usual Activity where N = 425

The baseline mean (SD) EQ-VAS score was 57.0 (21.7). Figure 2 shows the frequency of perceived problems in each of the five domains of the EQ-5D. The highest frequency of severe problems was observed in the pain/discomfort and anxiety/depression domains.

### Reimbursement

ExFOS enrolled patients reported, via questionnaire, whether the treatment would be reimbursed by public or private security. Reimbursement of teriparatide in Greece was almost exclusively covered by public health insurance. For this reason all patients went through a state committee that ensured treatment criteria were met in each and every case. During the 15 months of enrolment, which coincided with changes in the national reimbursement procedures, monthly enrolment varied from 11 to 45 patients per month. There was no evidence of enrolment variability associated with socioeconomic milestones during the study.

### Juxtaposing description ExFOS and EFOS cohorts at baseline

**Fig. 3:**
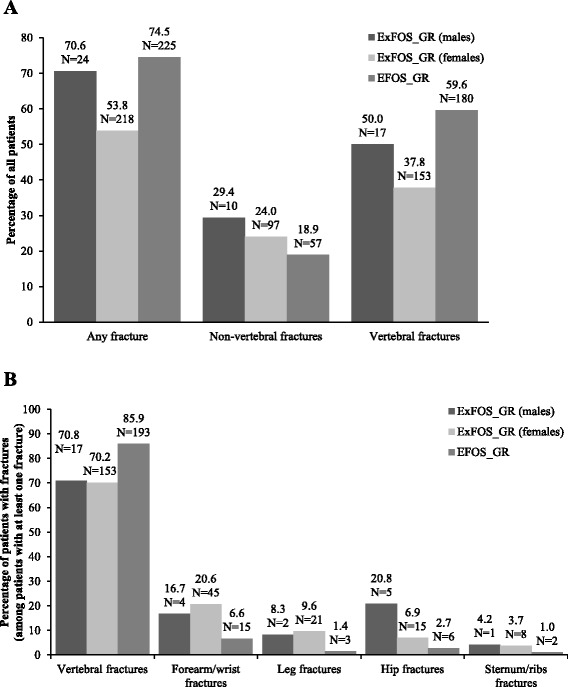
Short title: Location of previous fractures for Greek ExFOS and EFOS participants. Detailed legend: Location of previous fractures in the Greek ExFOS cohort (by gender) and Greek EFOS cohort for (**a**) all patients in the cohort and (**b**) for patients with at least one previous fracture

When descriptively analysed, the EFOS and ExFOS female Greek cohorts appear to have similar age, somatometric measurements and bone density measurements (Table 1). Both cohorts exhibited severe osteoporosis with a high risk for new fractures, and experienced relatively frequent and severe back pain and diminished quality of life. Women in the ExFOS cohort seemed to be active (with higher exercise rates) and less frequently needed to use their arms when standing up from a chair. Noticeably, a lower percentage of women in ExFOS reported a history of fracture compared with women in the EFOS cohort. A similar percentage of women in both studies had received no previous osteoporosis treatment (17–18 %). Among the women in the ExFOS cohort, prior use of calcitonin was lower and prior use of bisphosphonates higher, than that seen in the EFOS cohort. Fewer women in ExFOS had a history of glucocorticoid use than in EFOS. Figure 3 depicts fracture prevalence in EFOS and ExFOS female participants.

## Discussion

In the current analysis, we have attempted to address questions about prescribing habits for teriparatide in Greece in everyday clinical practice, as well as describing the baseline characteristics of the Greek ExFOS cohort. Greece enrolled the highest number of participants in ExFOS among the eight participating countries [19].

### From EFOS to ExFOS

Baseline findings of both observational studies suggested that Greek patients suitable for teriparatide treatment tended to be elderly, have severe osteoporosis and be at high risk of new fractures. They also had relatively frequent and severe back pain, which can affect mobility and consequently quality of life [20–22]. A descriptive comparison between the two studies (Table 1) suggests that women initiating treatment with teriparatide more recently (ExFOS) were relatively healthier, more active, exercised more frequently, could rise from a chair more easily and had fewer previous fractures than their counterparts from EFOS.

In Greece, teriparatide treatment is usually only initiated following prior treatment with anti-osteoporotic medications. While the use of prior osteoporosis medication was high in the total ExFOS cohort, a considerable proportion of patients (19.8 %) were treatment naïve, in common with the EFOS cohort (16.9 %). Since experience with all osteoporosis medications has increased in the period between these two studies and the insurance needs for teriparatide reimbursement include failure of prior osteoporosis treatments, we can only speculate that the treatment naïve patients have been first diagnosed with osteoporosis after a severe event, such as clinical fracture. Our observations indicate there has been a decrease in calcitonin use in recent years, which may reflect concerns of Greek health care practitioners concerning the benefits and risks of calcitonin-containing medicines. These concerns would have been manifest before the final recommendation of the European Medicines Agency (EMA) that calcitonin use should be limited for the treatment of osteoporosis (this recommendation was made in July 2012, 2 months after enrolment into ExFOS was completed, and stated that calcitonin-containing medicines should only be used for short-term treatment because of evidence that long-term use is associated with an increased risk of cancer, and further that they should no longer be prescribed as nasal sprays for the treatment of osteoporosis) [23].

The study enrolment rate appeared to increase by about 45 % in ExFOS compared to EFOS. This may be explained by the increased familiarity of health care practitioners with teriparatide, and its benefits and risks, and greater awareness of observational studies and the benefits of enrolment. Indeed, many sites were common in both Greek studies, which ameliorates a possible bias due to differing investigators’ perceptions in each study.

The proportions of patients enrolled without a history of fracture increased considerably between the two studies (in EFOS 15.9 % had confirmed no fracture history and 9.6 % did not provide data versus 46.2 % with no fracture history in the female Greek ExFOS subgroup); this discrepancy may be explained in part by changes to reimbursement criteria. A criterion for teriparatide use—only after sustained fracture—was in place during EFOS, but was removed after May 2007. Thus, in ExFOS, committees were able to approve treatment with teriparatide regardless of fracture history.

### Impact of changes in health reimbursement system on prescription habits

The Greek health system is complex and based on interplay between a national system financed by the state, and medical expenses financed by social insurance organisations for insured patients. Funding for health services and products comes primarily from the state budget and social insurance funds [24].

Teriparatide was almost exclusively reimbursed via public insurance institutes. Public reimbursement criteria as well as reimbursement coverage rates have been amended in many occasions during the ExFOS enrolment period.

Since the economic crisis caused a deterioration of health care [16, 17] and considering the problems faced regarding drug provision by private pharmacies, particularly for teriparatide we were anticipating enrolment delays, especially at milestones of important health care changes. Nevertheless, these factors did not negatively affect enrolment into ExFOS, since the enrolment rate pattern per month was similar to that of other observational studies performed before the economic crisis.

Results from ExFOS show that, at present, teriparatide-treated patients are largely elderly patients. As this segment of the population usually comprises insured pensioners, and since teriparatide is, currently, 100 % reimbursed after being approved by a specialized committee, few patients are currently affected financially. In addition, most patients suitable for teriparatide treatment still visit their healthcare practitioner. In the future, this picture could change. Indeed a growing portion of the Greek population no longer seek preventive care. Whether this will affect consulting patterns during ExFOS and the long-term outcomes of the study remains to be seen.

### Impact of EU license changes on prescription habits

The most important change in the EU label of teriparatide, the extension of teriparatide treatment for up to 24 months, has been incorporated into the ExFOS protocol. This longer duration of therapy has been associated with an increase in non-vertebral fracture protection and a reduction in back pain [25]. Observational studies, such as DANCE (Direct Assessment of Non-vertebral Fractures in the Community Experience), have shown that the reduction in the risk of non-vertebral fractures during 24 months of teriparatide treatment in men and women with osteoporosis stabilises during the last 6-month treatment period [26]. Furthermore, improvements in the severity of back pain and quality of life have also been recorded after 24 months of teriparatide treatment in postmenopausal women with osteoporosis [27]. ExFOS may provide data on the effect of this extended period of teriparatide treatment on the risk of fracture and the reduction in back pain.

Following the new indication for teriparatide of male osteoporosis*,* 34 Greek men (7.3 %) were enrolled in ExFOS; however, women still out-numbered men by more than 10-fold in the study cohort. This is accordance with Greek epidemiological studies, showing that the prevalence of osteoporosis is more than 10-fold higher in Greek women than in Greek men [28]. The number of treated Greek men in ExFOS could be perceived as lower than expected. However, despite causing significant morbidity and mortality [29], male osteoporosis is still under-diagnosed, even in those with vertebral deformities [30]. Male patients treated with teriparatide were also in the minority in other observational studies, which included a similar percentage of males to that in our study (ISSO study: 9.5 % [31], DANCE study: 9.6 % [32], Yu et al.: 9 % [33]). As reported by Wong et al., in the DANCE study, patient gender may influence a physician’s decision to initiate teriparatide therapy, despite a similar proportion of men and women having prior fragility fractures at baseline and comorbid conditions that increase the risk of fracture [32]. Frailty, low body mass index and inadequate response or intolerance to previous osteoporosis therapy were reasons for physicians prescribing teriparatide more often in women than in men, whereas chronic glucocorticoid therapy was given as a reason for initiating teriparatide more often in men than women [32]. Likewise, in our study population, twice as many men as women had a history of glucocorticoid use. These results suggest a preference for prescribing teriparatide in females, regardless of the baseline risk of fracture. However, it is also possible that men were less likely than women to consent to participate in an observational study of a widely perceived ‘female’ health problem, even though men are generally more likely than women to enrol in clinical trials [34].

In our cohort, only about half of the enrolled men had received prior osteoporosis treatment. It is possible that our study included patients (especially men) who sustained fractures in the past but were never treated for osteoporosis, since data support low osteoporosis treatment rates in patients with a prevalent fracture [35]. In addition, the observation that men had experienced almost triple the number of hip fractures experienced by women, may provide further support for the finding that, in this study, teriparatide was used in men with more severe osteoporosis and newly diagnosed osteoporosis already complicated by fractures. We should note, however, that the low number (n = 34) of the males in the study population could be challenging in some respects and is a major limitation of the current per gender subanalysis.

Data on the use of teriparatide in GIOP are not available for our population from ExFOS. It is estimated that 2.5 % of the elderly population aged 70–79 years in Britain are prescribed oral glucocorticoid therapy [36]. Our population has the same age range, yet past glucocorticoid use was reported in a higher percentage of patients (8.9 %). Interestingly, oral glucocorticoid use was reported in an even higher percentage of patients from the EFOS Greek cohort (Table 1). Nevertheless, we consider it unlikely that GIOP was an important factor for teriparatide treatment, at least in patients enrolled in the current ExFOS study. The lack of specific recommendations for teriparatide treatment in GIOP due to cost, the absence of long-term data (due to the restricted duration of administration), and the actual or perceived inconvenience of a daily injection for 24 months [36] may explain the apparent decrease in the numbers of these patients in a study (ExFOS) in which a specific relevant indication has been incorporated. However, we know from the full EFOS cohort [37] that for the 18.6 % of women who were glucocorticoid users at any time during the study and who were treated with teriparatide for up to 18 months, analysis of fracture data in 6-month segments revealed that the adjusted odds of fracture were significantly decreased during the last year of follow-up (i.e., during the two 6-month periods covering 24–36 months after teriparatide was discontinued) compared with the first 6 months of teriparatide treatment: an 81 % decrease in the 24 to <30-month period (p < 0.05), and an 89 % decrease in the 30 to < 36-month period (p < 0.05). In addition, the glucocorticoid user group in EFOS demonstrated significant reductions in back pain and improvements in quality of life during teriparatide treatment that were maintained after the drug was discontinued [37]. It will be interesting to see the impact of a longer duration of teriparatide treatment (i.e. 24 months) on this specific population in the ExFOS cohort.

Regarding back pain, which is a very common symptom in the elderly in the absence of osteoporosis, its evolution during treatment is of great interest to us, considering the favourable effects shown in EFOS [4, 5, 38] as well as other studies [39], in contrast to the lack of differences in back pain-related endpoints in a head-tohead trial with risendronate [40]. Since these patients seem to have lower rates of fractures at treatment initiation, it will be of interest to observe self-reported back pain validation as a consequence of teriparatide treatment.

## Conclusions

In conclusion, Greek patients prescribed teriparatide in ExFOS had severe osteoporosis with an increased risk of fractures and back pain. Baseline data from the female Greek cohorts of ExFOS and EFOS suggest that the two study populations are relatively homogenous and, as a result, can provide interesting insights regarding the use of teriparatide for the treatment of osteoporosis in the past (EFOS) and present (ExFOS). A constant prescribing profile for teriparatide has been enriched by a noticeable difference in fracture history between the two study cohorts, which follows National reimbursement criteria and may indicate a change towards prescribing of teriparatide for less severely affected patients. The economic crisis in Greece did not seem to affect patient enrolment into ExFOS. Data are interpreted in the context of an observational setting.

## Abbreviations

BMD: Bone mineral density
DANCE: Direct Assessment of Non-vertebral Fractures in the Community Experience
EFOS: European Forsteo Observational Study
EMA: European Medicines Agency
EOPYY: National Organization for Health Care Provision
EQ-5D: EuroQol-5 Dimension
EQ-VAS: EuroQol visual analogue scale
ExFOS: Extended Forsteo Observational Study
GIOP: glucocorticoid-induced osteoporosis
IMF: International Monetary Fund
SD: standard deviation
